# Percutaneous kyphoplasty through unilateral puncture on the convex side for the treatment of painful osteoporotic vertebral compression fracture with scoliosis

**DOI:** 10.1186/s12891-024-07399-w

**Published:** 2024-04-16

**Authors:** Qiuhan Li, Song Wang, Qing Wang, Pijun Yan, Jin Yang

**Affiliations:** 1 Department of Clinical skills center, Affiliated Hospital of Southwest Medical University, 25 Taiping Road, Luzhou, 646000 Sichuan China; 2 Department of Orthopedics Surgery, Affiliated Hospital of Southwest Medical University, 25 Taiping Road, Luzhou, 646000 Sichuan China; 3 Department of endocrinology, Affiliated Hospital of Southwest Medical University, 25 Taiping Road, Luzhou, 646000 Sichuan China

**Keywords:** Painful osteoporotic vertebral compression fractures, Scoliosis, percutaneous kyphoplasty, Unilateral puncture, Convex side

## Abstract

**Purpose:**

To assess the clinical safety, accuracy, and efficacy of percutaneous kyphoplasty (PKP) surgery using an enhanced method of unilateral puncture on the convex side for the treatment of painful osteoporotic vertebral compression fractures (P-OVCF) with scoliosis.

**Methods:**

Clinical and radiographic data of P-OVCF patients with scoliosis who underwent PKP via unilateral puncture on the convex side from January 2018 to December 2021 were retrospectively analyzed. This technique’s detailed surgical steps and tips were described. The local kyphosis angle (LKA), scoliosis Cobb angle (SCA), and local scoliosis Cobb angle (LSCA) were measured using X-ray and compared at pre-operation, post-operation, and the last follow-up. The width of pedicle (POW), inner inclination angle (IIA), lateral distance (LD), and puncture course length (PCL) were measured on the axial computed tomography image and compared between two sides. Postoperative computed tomography was employed to evaluate the condition of cement distribution and puncture. Clinical outcomes were evaluated using the Oswestry Disability Index (ODI) and Visual Analog Scale (VAS) for back pain (BP).

**Results:**

Thirty-six patients, 23 women and 13 men, with an average age of 76.31 ± 6.28 years were monitored for 17.69 ± 4.70 months. The median surgical duration of single vertebrae was 35 min. The volume of bone cement for single vertebrae was 3.81 ± 0.87 ml and the proportion of sufficient cement distribution of the patients was 97.22. LKA was considerably improved from pre-operation to post-operation and sustained at the last follow-up. SCA and LSCA were not significantly modified between these three-time points. IIA, PCL, and LD were lower on the convex side than on the concave side. POW was considerably wider on the convex side. The ODI and VAS-BP scores were significantly improved after surgery and sustained during the follow-up.

**Conclusions:**

Combining with the proper assessment of the pre-injured life status of patients, PKP surgery using unilateral puncture on the convex side for the treatment of P-OVCF with scoliosis can achieve safe, excellent clinical, and radiographic outcomes.

## Introduction

Percutaneous kyphoplasty (PKP), a minimally invasive operation, has been extensively used to treat painful osteoporotic vertebral compression fractures (P-OVCF) [1, 2] with lesser complications than percutaneous vertebroplasty [3, 4]. The key to a successful PKP surgery is a satisfactory puncture, which not only allows for symmetrical bone cement distribution but also helps to avoid complications [5, 6]. As technology has advanced, various intraoperative guiding methods for PKP puncture have been introduced, but PKP under C-arm fluoroscopy is still the most commonly used [7–10].

The unilateral puncture approach with less surgical time, cement consumption, a lower cement leakage ratio, and better short-term general health compared to bilateral puncture is recommended for PKP [10, 11]. The ideal target puncture point for a unilateral approach is thought to be in the vertebral body’s anterior one-third middle point [12, 13]. To make needles successfully reach the target point through the unilateral puncture, the proper inner inclination angle (IIA) and the distance from the puncture point to the midline in several puncture techniques were suggested in previous studies [13–17].

P-OVCF with scoliosis may have asymmetric vertebrae and pedicles in severely deformed and rotated spines, furthermore, the degeneration of facet joints, costotransverse joint, calcification shadow of costal cartilage and osteoporosis decrease the quality of intra-operation fluoroscopy to cause challenges for accurately located lesion vertebrae and puncture point. Although each technique has its inherent benefits, none guarantees safety and accuracy in this situation, and none is easily conducted by surgeons with little experience. Therefore, an accurate puncture for PKP is difficult for a rotated osteoporotic spine with asymmetric pedicles.

According to our knowledge, there was no research to describe a specific method to treat P-OVCF with scoliosis. Therefore, we presented an enhanced method of unilateral puncture on the convex side to conduct PKP surgery for the treatment of P-OVCF with scoliosis. This study seeks to describe and assess this method as to its clinical safety, accuracy, and efficacy.

## Materials and methods

### Study population and inclusion criteria

Between January 2018 and December 2021, 416 patients having P-OVCF who had PKP surgery in our department were regarded as a candidate for screening. Based on the following inclusion and exclusion criteria: (1) P-OVCF patients with scoliosis (P-OVCF was determined by typical clinical presentation and MRI demonstration; scoliosis was defined as the Cobb angle more than 10°); (2) the lesion vertebrae located in the curve of scoliosis; (3) using unilateral puncture on the convex side; (4) osteoporosis ( ≤ − 2.5 T-score measured using dual-energy X-ray bone mineral density scan);5) patients with complete medical records and radiographic data for more than 12 months;

Exclusion criteria: (1) patients with chronic mechanic back pain (BP) for more than 1 year (Visual Analog Scale [VAS] > 4); (2) patients with pathologic vertebral lesions including vertebral metastatic carcinoma, vertebral hemangioma, and myeloma; (3) patients with neurological deficits caused by spinal cord or nerve root compression. This retrospective study was authorized by Ethics Committee of the Affiliated Hospital of Southwest Medical University, and strictly followed the guidelines of the Declaration of Helsinki (No. KY2023024). All patients finally included in this study signed informed consent.

### Surgical technique

**Fig. 1:**
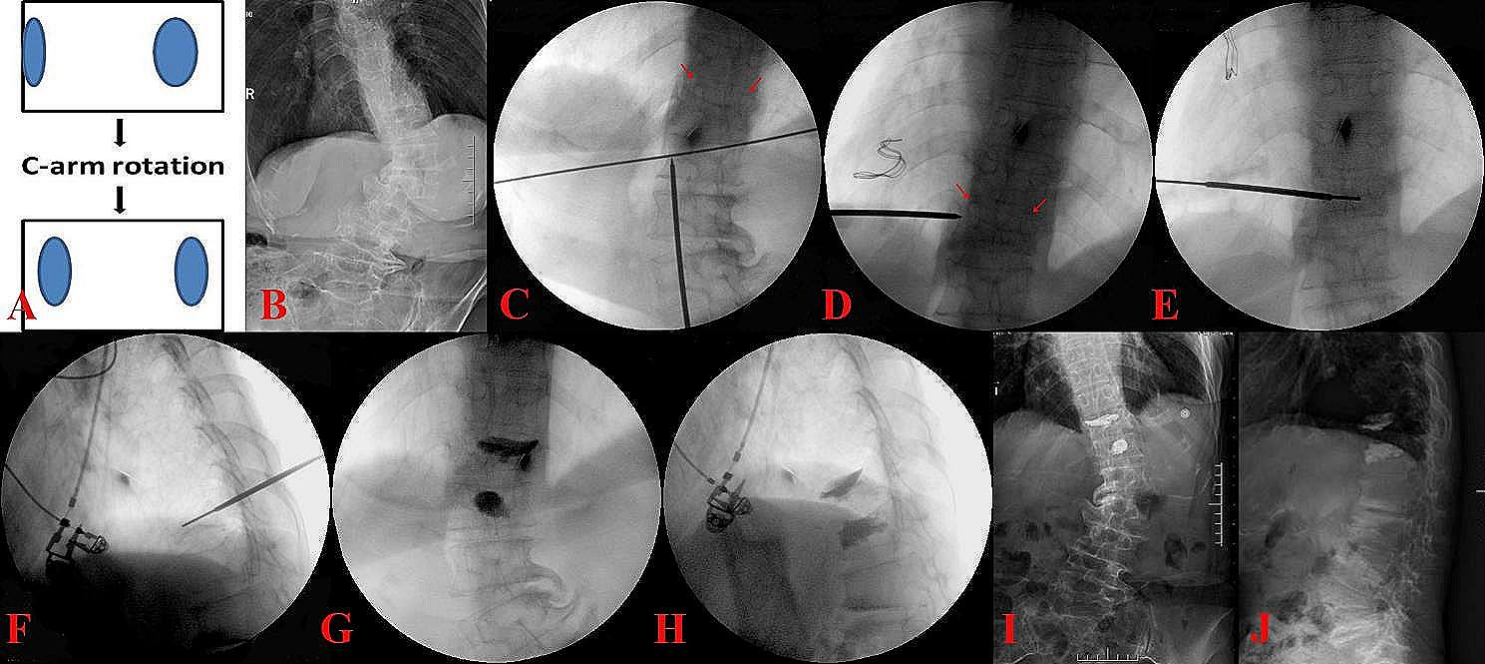
**A**, C-arm is gradually rotated until a true PA image of the rotated vertebral body is obtained and both pedicles are mostly symmetrically visualized en face; **B**, the X-ray of PA view at pre-operation; **C**, the intraoperative PA image revealed asymmetrical pedicle shadows (red arrows); **D**, C-arm position is modified and gradually rotated to obtain a true PA image of the rotated vertebral body and both pedicles are nearly symmetrical (red arrows); E and F, the guide wire was at or cross the midline in PA view (**E**) and reached the anterior one-fourth of the vertebral body at the lateral view (**F**); G and H, the final intraoperative PA (**G**) and lateral (**H**) images; I and J, the PA (**I**) and lateral (J) X-ray after surgery

**Fig. 2:**
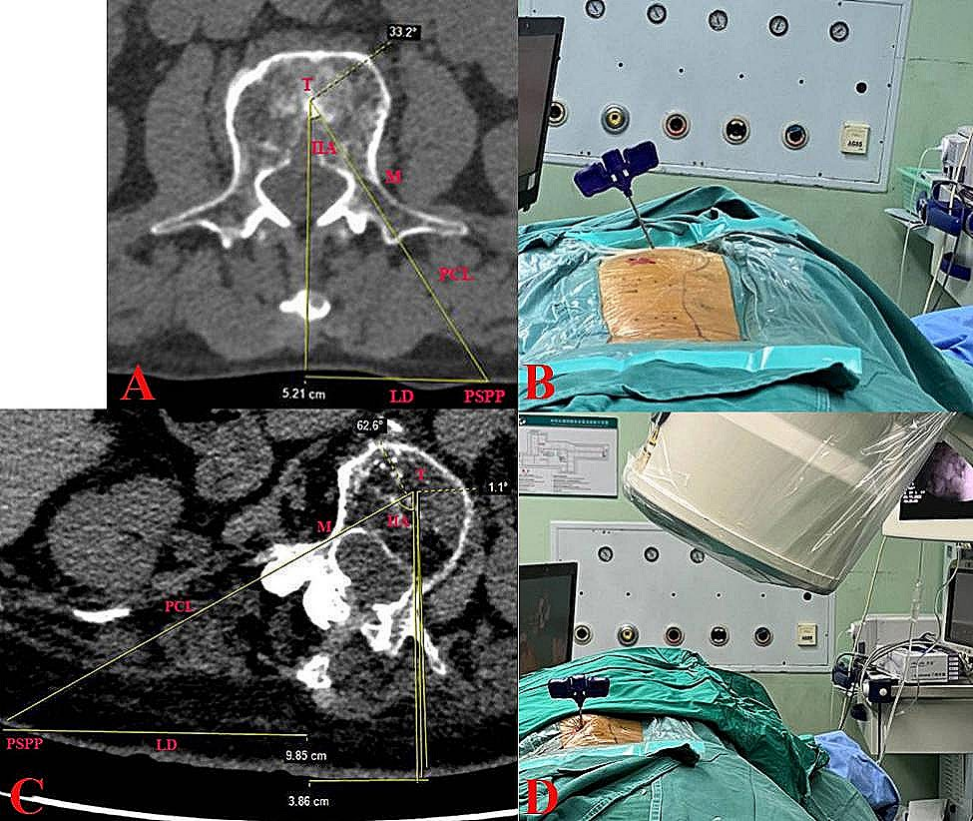
A, a schematic diagram of standard unilateral puncture, in which IIA was 33.2° and the LD was 5.21 cm; B, the IIA of puncture on non-rotated vertebrae; C, a schematic diagram of unilateral puncture on rotated vertebrae, in which IIA was 1.1° on the convex side and 62.6° on the concave side, respectively; LD and PCL were lower on the convex side than the concave side; D, the C-arm was rotated, and the puncture was nearly vertical (IIA was 1.1°). T, target point; M, midpoint of the pedicle; PCL, puncture course length; PSPP, planned skin puncture point; LD, the lateral distance between PSPP and the spinous process; IIA, inner inclination angle

The patient is kept in a prone position and routinely disinfected and the operation was conducted under general or local anesthesia. First, a C-arm fluoroscope is placed at the target vertebra for a poster-anterior (PA) image. The C-arm is gradually rotated until a true PA view of the rotated vertebral body is achieved and both pedicles are approximately symmetrically visualized (Fig. 1A–D). Once the symmetrical pedicle shadows are obtained, the C-arm is rotated in the sagittal plane to try to create a tangential view of both upper and lower endplates. The puncture point was selected at the skin location which ranged from 2.4 to 5.15 cm lateral to the midline on the convex side based on the target vertebrae level. The puncture was performed via a transverse process-pedicle approach [13], in the thoracic spine via the costotransverse joint. The IIA of puncture on the convex side was clearly lower than that on the concave side (Fig. 2). Based on the oval pedicle outline on intraoperative fluoroscopy, the entry points are determined at 9-o’clock and 3-o’clock positions on the left and right pedicles, respectively (Fig. 1D). When the needle tip reached the midpoint of the pedicle in the PA view, the C-arm position was changed to obtain a true lateral view. We can now observe and adjust the inclination angle of the sagittal view in the lateral view, as well as the IIA of the puncture. After making corresponding judgments and adjustments, the C-arm position was changed to obtain a true PA view. The puncture was continued until the needle tip reached the pedicle’s medial margin. The C-arm position was changed to obtain a true lateral view, and the puncture was considered ideal if the needle tip was located in the posterior one-fourth of the vertebral body. The guide wire was inserted to reach the anterior one-fourth of the vertebral body in lateral view and was at or crossed the midline in PA view (Fig. 1E, F). The expansion cannula, working cannula and inflatable balloon were inserted sequentially and expanded under fluoroscopy guidance. The prepared bone cement is slowly inserted and stopped as the cement reached the posterior one-sixth of the posterior wall of the vertebra [18]. After the bone cement penetrates and distributes well in the fractured vertebral body, the cannula is removed and the operation is finalized (Fig. 1G–J). All patients were given anti-osteoporosis medication after the operation and got out of bed on the day after the operation and were advised to avoid extreme physical strain for 3 months.

**Fig. 3:**
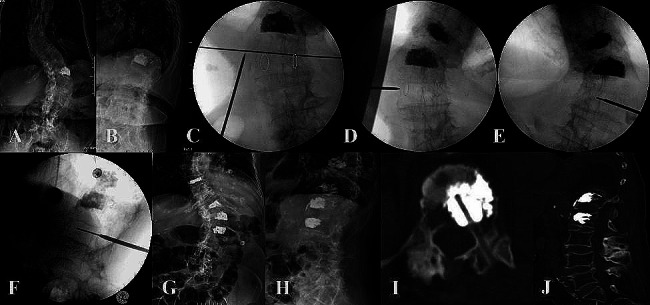
**A** and **B**, the X-ray of PA and lateral view at pre-operation; **C**, the intraoperative PA image revealed asymmetrical pedicle shadows; **D**, C-arm position is rotated and both pedicles are nearly symmetrical; E and F, the guide wire was at or cross the midline in PA view (**E**) and reached the anterior one-fourth of the vertebral body at the lateral view (**F**); G and H, the PA (**G**) and lateral (**H**) X-ray after surgery; **I**, the axial CT image revealed adequate cement distribution and the puncture track; **J**, the sagittal CT reconstruction image revealed the vertebral height and sufficient cement distribution

A representative case is shown in Fig. 3.

### Evaluation methods and parameters

#### Clinical efficacy parameters

Patients’ subjective BP perception was evaluated by VAS. The patient’s quality of life was evaluated using Oswestry Disability Index (ODI) scores. These parameters were examined by two independent observers before surgery, 1 day after surgery, and at the final follow-up. Furthermore, as these OVCF patients with scoliosis and some had noticeable sagittal and or coronal imbalance of the whole spine, we further assessed the living quality before the acute BP onset and regarded it as a critical reference of our treatment goal.

#### Radiographic parameters

The X-ray was conducted before surgery, 1 day after surgery, and at the final follow-up. The local kyphosis angle (LKA) of the injured vertebral body in the lateral X-ray was measured to assess the extent of vertebral damage and restoration and its improvement after surgery. LKA was the angle formed by lines drawn parallel to the upper endplate of the upper adjacent vertebrae and the lower endplate of the lower adjacent vertebrae. The scoliosis Cobb angle (SCA) and the local scoliosis Cobb angle (LSCA) in the PA view were documented to assess the difference between pre-operation and post-operation. SCA was the angle of scoliosis in which the lesion vertebrae were involved. LSCA was the Cobb angle formed by lines drawn parallel to the upper endplate of the upper adjacent vertebrae and the lower endplate of the lower adjacent vertebrae. The vertebral rotation was examined by the method proposed by Nash-Moe [19].

The 256-line multi-slice CT-Scanner (Siemens SOMATOM Definition Flash, Siemens Healthcare, Germany) was conducted before surgery, and at 1 day postoperatively for all patients, providing 1-mm thick axial helical with three-dimensional reconstructions. The width of pedicle (POW), IIA, the lateral distance (LD) between the planned skin puncture point (PSPP) and the spinous process, and the puncture course length (PCL) at convex and concave sides of the target vertebrae were documented and compared. In the axial CT view, the cross-point between the anterior one-third of the vertebral body and the midline of the body was the target point (T); the middle point of the pedicle was presented as point M. PCL was the line from point T to M and its extended line to the skin. PSPP was the cross-point between PCL and skin (Fig. 2).

The cement distribution, cement leakage, and puncture track were assessed. The criteria for cement leakage were divided into five types: type B (through the basivertebral vein), type S (through the segmental vein), type C (through a cortical defect), and type D (intradiscal leakage)], and mixed type (two or more types existing [20]. Insufficient cement distribution was the distribution of less than half of the fracture area of the index vertebra demonstrated on postoperative axial and sagittal or coronal CT scans [21]. The axial CT images were used to examine the puncture tract to investigate if the pedicle medial wall is intact.

All radiographic parameters were evaluated three times at multiple time points by two independent observers (2 senior spinal surgeons). Any disagreements for non-quantitative parameters between the observers were addressed by reassessment and consensus.

### Statistical analysis

SPSS (version 24.0, IBM Corp., Armonk, NY, USA) was used for data analysis in this study. The Shapiro–Wilk test was employed for testing the normality of data. Continuous data were presented as mean and standard deviation when normally distributed or as median and interquartile range when not normally distributed. Continuous data were assessed using a Student t-test for parametric data and a Mann–Whitney U test for nonparametric data. The repeated-measures data were assessed using repeated-measures analysis of variance and the Bonferroni correction for multiple comparisons. *P* < 0.05 signified statistical significance.

## Results

### Demographics characteristics and surgical data

Thirty-six patients, 23 women, and 13 men, with a mean age of 76.31 ± 6.28 years were enrolled and examined. The follow-up period was 17.69 ± 4.70 months. The duration of symptoms was 21.25 ± 12.42 days. The body mass index was 22.97 ± 2.90 and the median bone mineral density was minus 3.6. The surgical levels were T5–T10 in 4, T11–L2 in 26, and L3–L5 in 6 patients. One-vertebrae PKP surgery was conducted in 8 patients, 2-vertebrae in 22, and 3-vertebrae in 6. Thirty-four patients underwent local anesthesia and the remaining two had general anesthesia. There were 11 patients with rotation grade 1, 19 with grade 2, and 6 with grade 3 according to the classification of Nash-Moe [19]. The median surgical duration of single vertebrae was 35 min (IQR, 30–40 min). The mean intraoperative fluoroscopy of single vertebrae was 39.43 ± 5.40 times. The volume of bone cement for single vertebrae was 3.81 ± 0.87 ml and the proportion of sufficient cement distribution of the patients was 97.22. The postoperative hospital stay was 2.67 ± 0.89 days. Table 1 detailed more information.

**Table 1:** Demographic characteristics of 36 patients

Characteristics	Median (Interquartile Range) or Mean ± Standard Deviation (Range) or n (%)
Age(years)	76.31 ± 6.28
Gender(female, n,%)	23 (63.89)
Body mass index	22.97 ± 2.90
Bone mineral density(minus)	3.6 (4.07–3.20)
Duration of symptoms (days)	21.25 ± 12.42
Operated vertebrae (n,%)	
One	8 (22.22)
Two	22 (61.11)
Three	6 (16.67)
Location of fracture vertebrae (n,%)	
T5-T10	4 (11.11)
T11-L2	26 (72.22)
L3-L5	6 (16.67)
Scoliosis (n,%)	
Degenerative (including multiple asymmetric OVCF)	32 (88.89)
Adolescent idopathic combined with degenerative	4 (11.11)
Rotated degree of the operated vertebrae (n,%)	
Degree 1	11 (30.55)
Degree 2	19 (52.78)
Degree 3	6 (16.67)
Anesthesia (n,%)	
Local	34 (94.44)
General	2 (5.56)
Median operated time for single vertebrae (mins)	35 (30–40)
Mean fluoroscopic times for single vertebrae	39.43 ± 5.40
Mean volume of bone cement for single vertebrae (ml)	3.81 ± 0.87
Cement distribution sufficient (n,%)	35 (97.22)
Complications (n,%)	
Cement leakage	7 (19.44)
Puncture complication	2 (5.56)
Adjacent level vertebral fracture	2 (5.56)
Other complications	1 (2.78)
Hospital stay (days)	2.67 ± 0.89
Follow-up period(months)	17.69 ± 4.70

T: thoracic; L: lumbar; OVCF: osteoporotic vertebral compression fracture; Rotated degree of the operated vertebrae based on the Nash-Moe Method; mins: minutes; ml: milliliter

### Clinical and radiographic results

**Fig. 4:**
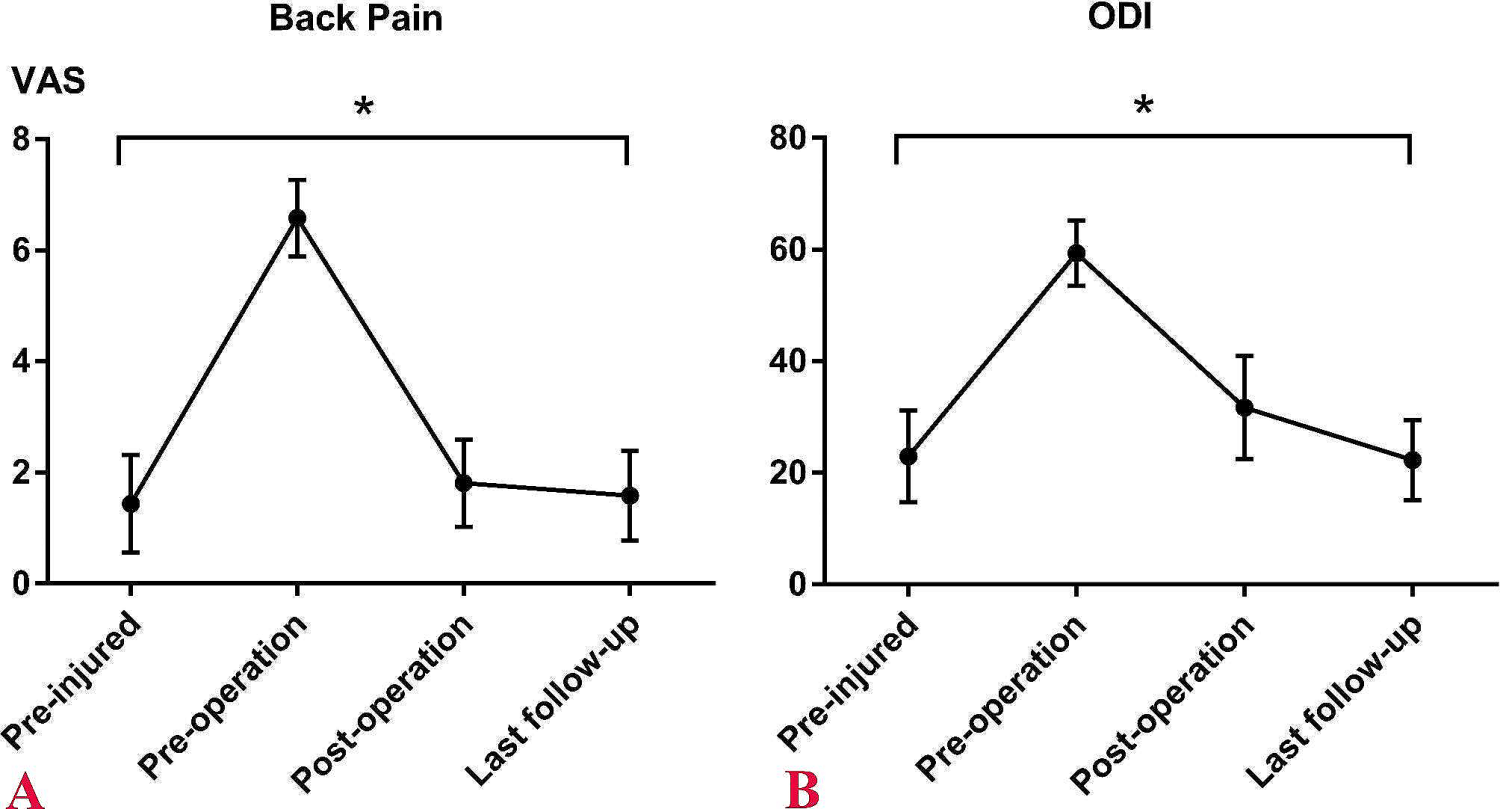
A Visual Analog Scale scores for back pain at the pre-injured, pre-operation, post-operation, and last follow-up assessments; B, Oswestry Disability Index scores at the pre-injured, pre-operation, post-operation, and last follow-up evaluations; * indicates statistical significance between preoperative score and score of any other time point

The mean pre-injured BP VAS score was 1.44 ± 0.88, which elevated to 6.58 ± 0.69 at pre-operation and improved to 1.81 ± 0.79 after surgery, and 1.58 ± 0.81 at the last follow-up (*P* = 0.000; Fig. 4A). The mean pre-injured ODI was 22.94 ± 8.23, which rose to 59.36 ± 5.84 at Pre-operation, and improved to 31.72 ± 9.30 after surgery, and 22.33 ± 7.24 at the last follow-up (*P* = 0.000; Fig. 4B).

LKA considerably improved from 17.00 ± 3.00 preoperatively to 12.00 ± 2.39 after surgery, and 12.38 ± 2.54 at the last follow-up (*P* = 0.000). SCA and LSCA were not substantially modified between these three-time points (*P* > 0.05). Table 2 detailed additional information.

**Table 2:** The comparison of radiographic parameters for the target vertebrae between pre- and post-operation

	Pre-operation	Post-operation	The last follow-up	*P* value	*F* value
Local kyphosis angle	17.00 ± 3.00^*^	12.00 ± 2.39^#^	12.38 ± 2.54	0.000	39.47
Scoliosis Cobb angle	20.64 ± 4.68	20.21 ± 4.92	20.35 ± 6.25	0.943	0.06
Local scoliosis Cobb angle	11.70 ± 3.18	11.39 ± 3.24	11.41 ± 3.76	0.913	0.09

^*^, a statistical significance between pre-operation, post-operation and the last follow-up; ^#^, no statistical significance between post-operation and the last follow-up; cm: centimeter

The POW of the injured vertebrae was considerably wider on the convex side than the concave side (0.62 ± 0.17 cm vs. 0.52 ± 0.11 cm, *P* = 0.003). IIA was considerably smaller on the convex side than the concave side (12.64° ± 8.04° vs. 50.15° ± 9.63°, *P* = 0.000). PCL was considerably shorter on the convex side than the concave side (7.37 ± 1.27 cm vs. 10.95 ± 2.26 cm, *P* = 0.000). Similarly, LD was shorter on the convex side than on the concave side (3.82 ± 0.75 cm vs. 6.70 ± 1.74 cm, *P* = 0.000). Table 3 showed detailed information.

**Table 3:** The comparison of puncture-relevant parameters between the convex side and concave side of the target vertebrae

	The width of the pedicle (cm)	The inner inclination angle	The puncture course length (cm)	LD (cm)
Convex side	0.62 ± 0.17	12.64 ± 8.04	7.37 ± 1.27	3.82 ± 0.75
Concave side	0.52 ± 0.11	50.15 ± 9.63	10.95 ± 2.26	6.70 ± 1.74
*P* value	0.003	0.000	0.000	0.000
*t* value	3.12	-17.94	-8.29	-9.12

LD: the lateral distance between planned skin puncture point and the spinous process; cm: centimeter

### Complications

Cement leakage (7 patients, 19.4%) was the main complication and fortunately, it did not lead to severe consequences. Among them, three patients with type D leakage, two with type C leakage, one with type S leakage, and one with mixed leakage (type C + D). These patients without any discomfort symptoms received no additional treatment and had a careful follow-up. Puncture complications occurred in two patients and did not cause neurological injury. The inner wall of the pedicles was a break and the puncture course slightly intruded into the spinal canal according to the postoperative CT outcome. The adjacent level vertebral fracture occurred in two patients (Table 1).

## Discussion

To the best of our knowledge, this is the first research to treat P-OVCF patients with scoliosis using PKP surgery through unilateral puncture at the convex side. Safe and effective results of this approach were observed for an average of 17 months of follow-up.

### Treatment goal and surgical decision

Clinically, P-OVCF or scoliosis can cause BP separately; sometimes the symptoms of BP are induced by both two. It is difficult to differentiate which is the main cause of BP from the aspect of symptomatology, however, by combining medical history with MRI, we can extensively clear the responsibility of BP. Patients with scoliosis may have BP or not. The BP symptoms resulting from scoliosis always reveal a chronic course and accumulated progression and have characteristics of mechanic BP due to the unstable spine [22, 23]. However, patients with P-OVCF are usually identified with acute or sub-acute BP, and most of them had a mild injury history such as fallen [24]. Therefore, careful medical history taking is critical to assess the life status of the P-OVCF patient with scoliosis before the acute BP onset to try to clear which is the main cause of BP?

An exact understanding of patient healthy expectations and acquiring collaboration with patients are important for surgeons. For patients with mild BP before acute BP onset, conducting a PKP to lower the acute pain may be the rational treatment, and scoliosis which did not induce severe BP underwent careful follow-up may be sound. For patients with chronic moderate to severe BP before acute BP onset, both P-OVCF and scoliosis may be the causes of symptoms, only PKP surgery may be insufficient to alleviate the patient’s symptoms, however, open surgery should be extra careful with these aging patients. These patients usually had several coexisting medical conditions and a high risk of open surgery [25, 26]. Therefore, the rational treatment goal and proper minimally invasive surgical method were equally critical to balance and achieve satisfactory clinical results and perioperative safety [27].

In this study, a careful assessment of the life status of the patients before the acute BP onset was conducted. The BP and ODI outcomes between pre-injured and pre-operation showed that the life status of the patients is tolerable (Fig. 4). Therefore, we presume that the acute BP was mostly due to the P-OVCF and PKP surgery can relieve the BP. Importantly, the patients should be clear that the acute BP was the ultimate treatment goal, and correction for scoliosis was unnecessary this time and should undergo careful follow-up.

### Unilateral puncture in convex side

The unilateral puncture method for PKP to treat P-OVCF in our center was last for over 10 years and excellent clinical outcomes were obtained [13, 28]. In clinical practice, we discovered that in P-OVCF patients with scoliosis, the puncture conducted on the convex side had several benefits including shorter PCL, lesser IIA, and bigger POW. These advantages would make the puncture simpler to achieve the target point and symmetrical bone cement distribution. Furthermore, the spinal cord or dural sac was usually on the concave side. Therefore, the puncture on the convex side may be easier and safer. The current study findings proved the aforementioned advantages and demonstrated safe and effective outcomes (Table 3; Fig. 4) which was even similar to that of prior studies including patients without scoliosis [9, 10, 17].

Cement leakage occurred in seven patients (19.4%) and puncture complications occurred in two patients (5.6%) in this research without severe consequences. The cement leakage complication rate was less than that of previous reports which ranged from 4.8 to 39% [17]. Despite the high rates, works of literature reported that only a few cases are symptomatic or have related complications [17]. The success rate of puncture in this study was 94.4%, which revealed the high safety of this puncture method and the findings were even similar to a previous study in which the puncture was assisted by a robot with success rates ranging from 92.9 to 100% [8].

However, being unable to correct scoliosis or the likelihood of causing severer scoliosis was a theoretical disadvantage of this puncture in contrast with a bilateral puncture or unilateral puncture through the concave side. PKP through the concave side has the possible advantage of correction in a patient with scoliosis, however, this correction ability may be weak for the whole spine and followed by higher technique challenges and risks (Table 3). This study’s outcomes revealed that after achieving a satisfactory puncture and symmetrical bone cement distribution in these patients, severer scoliosis was not observed after surgery, and SCA and LSCA were not statistically substantial between pre-operation, post-operation, and the last follow-up (Table 2). Same to previous studies [4, 7, 17], the LKA was enhanced significantly after surgery (Table 2). This enhanced sagittal alignment of the spine combined with anti-osteoporosis drug treatment may reduce the risk of re-fracture of the spine to some degree.

### Technical notes

Asymmetric, rotated vertebrae and pedicles combined with osteoporosis prevent visualization of round pedicle shadows. It is difficult to conduct an accurate puncture into such pedicles, which have a highly variable and complex three-dimensional orientation. Furthermore, small pedicles are often found in scoliosis, particularly on the concave side, study reported that the proportion of extremely small pedicles was 7.8% [29]. Therefore, two technique notes were critical in PKP surgery for P-OVCF patients with scoliosis. The first is to obtain symmetrical or nearly symmetrical pedicle shadows on a true PA view via a rotating C-arm fluoroscope. The second was to conduct a puncture on the convex side. These procedures allow surgeons to carry out the PKP surgery under relatively high-quality visualization, with lesser anatomical difficulties, and technique issues.

### Limitations

This study has several limitations. First, the retrospective study may have presented selection bias. Second, anterior vertebral height was not included in the assessment parameters, for the height was unable to accurate measurement on the X-ray in scoliosis. Finally, the follow-up period of this study was relatively short and the time point of the last follow-up was different due to patient compliance.

## Conclusion

This study presented an enhanced method of unilateral puncture on the convex side to conduct PKP surgery for the treatment of P-OVCF with scoliosis. In combination with adequate assessment of the pre-injured life status of patients, this technique for P-OVCF with scoliosis can achieve safe, excellent clinical, and radiographic outcomes.

## Data Availability

The datasets in the current study are available from the corresponding author on reasonable request.

## Abbreviations

BP: back pain
IIA: inner inclination angle
SCA: scoliosis Cobb angle
LD: lateral distance
LSCA: local scoliosis Cobb angle
LKA: local kyphosis angle
ODI: Oswestry disability index
PA: posterior-anterior
PCL: puncture course length
PKP: percutaneous kyphoplasty
P-OVCF: painful osteoporotic vertebral compression fractures
PSPP: planned skin puncture point
POW: width of pedicle
VAS: visual analog scale
